# The Hospital Officer's Bookshelf

**Published:** 1912-03-02

**Authors:** 


### THE HOSPITAL OFFICER'S BOOKSHELF.

Health and Disease. (The Home University Library.) By W. Leslie Mackenzie, M.D., D.P.H., F.R.C.P. (London: Williams and Norgate. 1911. Price 1s. net.)

The steady drift from curative to preventive medicine is concisely and vigorously related by Dr. W. L. Mackenzie in this volume of the Home University Library. The book is one we can heartily recommend, not only to all institutional workers, but also to medical men. The author's style is at once crisp and convincing, and the matter is well chosen. We know of no other small book in which the many problems pertaining to the health of the community at large are discussed so pleasantly and instructively for the benefit of the ordinary reader. Such matters as the administrative aspects of tuberculosis, disease, and destitution, immunity, antitoxin, milk-supply, and epidemics are dealt with in a fashion that cannot fail to enlighten public opinion and help materially to produce that attitude of sympathy so necessary for the furtherance of preventive medicine and the evolution of the health movement. The chapter on the tubercular diathesis is full of interest on account of the trenchant criticism which certain views on this subject receive at the hands of Dr. Mackenzie. The book includes a brief exposition of the Insurance Bill, and is also in other respects thoroughly up to date. We shall be glad to hear that this little volume has had a wide sale.

The Tobacco Habit: Its History and Pathology. By Herbert H. Tidswell, M.R.C.S., L.R.C.P. (London: J. and A. Churchill. 1912. Pp. 246. Price 3s. 6d. net.)

Considering the earnestness with which the author has so obviously approached his subject, it becomes an unpleasant duty to record that this work is of no value as a contribution to the scientific study of the tobacco habit. In his biassed outlook the author thoroughly overreaches himself, and his so-called appeal must of necessity fail in its objective. With courageous yet ill-directed vigour, Dr. Tidswell states his opinion that "it would be better for a young man to be deprived of the advantages of a University than to run the risk of acquiring one of the worst habits of modern times"—namely, the "filthy habit" of smoking tobacco! Throughout the book one's eyes are assailed by the crudest of pseudo-scientific evidence and illogical deductions; while moral questions are dragged in irrelevantly to an absurd extent. This appeal "to medical students and to all members of the medical profession who are true Christians, etc.," is one which is not only a foredoomed failure, but one the spirit of which is not unlikely to meet with resentment in its present guise. The sub-title of the book, "A Study in Birth-rates: Smokers Compared with Non-Smokers," is the excuse for the inclusion of a large number of quotations which the author fondly imagines deserve the careful consideration accorded only to the most authoritative scientific papers.

Three Thousand Years of Mental Healing. By G. B. Cutten, M.D. Illustrated. (London: Hodder and Stoughton. 1911. Price 6s. net.)

The term "mental healing" is used by the author of this interesting book in the broadest possible sense. It comprehends for him any cures which may be brought about by the influence of the mind over the body, regardless of whether the power at the back of the cure is supposed to be a deity, a demon, another human being, or the mind of the patient himself. The subject is dealt with historically, from the earliest Babylonian, Egyptian, and Homeric times onwards. The faith-healers of the last century are dealt with in the concluding chapter, but the greater part of the work is devoted to the evolution of mental healing as exemplified by the connection of therapeutics in the various Christian Churches. A special chapter deals with the Royal Touch for the King's Evil. Mesmer naturally receives a fair share of attention, and the number of other charlatans of the eighteenth century who receive notice is very considerable. He asserts, indeed, that the wheat has now largely been sifted and the chaff thrown away, though he adduces little evidence in support of this confident and too optimistic statement. The amazing developments of Eddyism and the thousand-and-one similar fads and freaks of American hysterico-psychasthenic neurosis are briefly mentioned. As a record of human folly and credulity, the list is one which later ages will regard with amused scorn; as a commentary on our boasted civilisation it is a very wholesome corrective. One leaves this book more than ever impressed with the truth of Carlyle's sarcastic description of the inhabitants of Britain, and with the strong conviction that it applies with even greater force to the inhabitants of some other countries, certainly the United States.

The Fourth Report of the Wellcome Tropical Research Laboratories at the Gordon Memorial College, Khartoum. (Baillière, Tindall, and Cox. Vol. A., Medical. 1911. 21s. net.)

Of the great names in tropical research there is perhaps none which stands for such versatility, such devotion to hard work, and such ability to organise and to inspire the work of others, as that of Dr. Andrew Balfour. Nowadays he is known far and wide as the indefatigable director of the Khartoum Research Laboratories, as the author of innumerable papers on tropical diseases and parasites, and the producer of a series of reports which are second to none in value, in arrangement, in artistic unity and excellence. Yet it is not much more than a decade ago since Balfour the Rugby International, and Balfour the romantic novelist submerged their identity in the strenuous career and exile of an investigator of disease in the Sudan.

The Fourth Report, now issued, contains the discoveries and observations of the three years which have elapsed since the third one was issued. Like its predecessors, it is superbly illustrated and beautifully printed. Space does not allow of a detailed criticism of this monumental work; but if there is one paper more than another which ought to be singled out for mention, it is that of the Director upon the fallacies and puzzles of blood examinations. The coloured illustrations showing blood platelets overlying red corpuscles and similar traps for the unwary are admirable examples of the sort of work which the medical profession has come to expect in these reports.

Of the more economic aspect of the work with which the Report deals there is much that could be said. The papers dealing with the problems of sleeping sickness and kala-azar in the Anglo-Egyptian Sudan are good evidence of the way in which the white man's burden is being shouldered in those remote outposts of the Empire. To express the conviction that the work of the Wellcome Laboratories is an honour, not merely to the Gordon Memorial College, but to Great Britain and to civilisation, is no figure of speech, but a tribute which those who read this Report and its predecessors will find to be well deserved.

